# Onion Essential Oil-in-Water Emulsion as a Food Flavoring Agent: Effect of Environmental Stress on Physical Properties and Antibacterial Activity

**DOI:** 10.1155/2022/1363590

**Published:** 2022-10-05

**Authors:** Elham Taghavi, Afifah Syazwani Abdul Salam, Navideh Anarjan, Elexson Nillian, Mohd Nizam Lani

**Affiliations:** ^1^Faculty of Fisheries and Food Science, Universiti Malaysia Terengganu, 21030 Kuala Nerus, Terengganu, Malaysia; ^2^Department of Engineering, Tabriz Branch, Islamic Azad University, Tabriz, Iran; ^3^Faculty of Resource Science and Technology, Universiti Malaysia Sarawak, 94300 Kota Samarahan, Sarawak, Malaysia

## Abstract

Plant essential oils (EOs), which are acknowledged as generally recognized as safe (GRAS) by the Food and Drug Administration (FDA), have the potential to be used as a flavoring agent. However, there are limitations to some EOs, such as low water solubility and high volatility, which limit their application in food technology. This study was conducted to develop onion (*Allium cepa*) EO as a flavoring agent and determine its stability against environmental stress via an emulsification technique, with different concentrations of sodium caseinate, as a delivery system. Emulsions containing onion EO were prepared using different concentrations of sodium caseinate (3, 5, and 7% *w*/*w*) via the solvent-displacement technique. The physical properties (average droplet size, color, turbidity, and stability measurement) and antibacterial activity (agar disk diffusion method) of emulsions were then determined. Results show that emulsion with 7% (*w*/*w*) sodium caseinate was the most desirable sample in terms of physical properties and antibacterial activity. Hence, it was selected for environmental stress studies (i.e., thermal processing, freeze-thaw cycles, and ultraviolet (UV) exposure). Results revealed that all types of environmental stresses had significant (*p* < 0.05) effects on droplet size, color, turbidity, and stability. Generally, the environmental stresses increased the droplet size except in the freeze-thaw cycle case, while all stresses decreased the stability and lightness. All types of environmental stress treatment did not show a significant (*p* < 0.05) effect on antibacterial activity enhancement against *Salmonella* Typhimurium and *Listeria monocytogenes* except in the case of UV treatment against *L. monocytogenes*. Therefore, the present work has demonstrated the potential use of emulsion as an encapsulation and delivery system of EO flavors for food applications.

## 1. Introduction

Flavors have an important role in the consumer acceptability, palatability, and quality of food in the food industry. A first judgment about the value of a food source is made on its appearance and smell [1]. On the other hand, due to increasing mankind's knowledge, people look for safe food with minimum side effects. Not surprisingly, consumers express considerable concern about the indiscriminate use of chemicals in foods (i.e., synthetic flavor materials) [2]. Essential oil (EO) is considered a natural flavoring [3] and has been used for centuries as perfume fragrances, in culinary as a flavoring, and in folk medicine [4]. For most purposes, EO is the preferred flavoring agent and is commercially available [2].

The use of EOs as flavoring agents is registered by the European Commission (EC) and by the FDA, and they are classified as GRAS (under section 201 (s) and 409 of the Act and FDA's implementing regulations in 21 CFR 182.20) and approved in the food additive status list [5, 6]. Onion oil is the non-water-soluble fraction from the steam distillation of macerated onions [7]. Also, onion flavors (EO) are important seasonings widely used in food processing [8]. Recent research has demonstrated that onions possess several properties, such as antibacterial [9], antimicrobial (bacteria, molds, and yeasts) [8] antimutagenic [10], and antioxidant activities [8, 11]. The most medicinally significant components of onion oil are the organosulfur-containing compounds [12, 13]. These compounds are reactive, volatile, odor producing, and lachrymatory [14]. The bioactive properties and characteristic flavor of onion have been attributed to sulfur-containing compounds, which are the main constituents of its EO (dipropyl disulphide (21.31-60.4%), dipropyl trisulphide (17.1-21.92%), methyl 5-methylfuryl sulphide (18.3%), methyl 3,4-dimethyl-2-thienyl disulphide (11.75%), methyl 1-propenyl disulphide (13.14%), methyl 1-propenyl trisulphide (13.02%), methyl propyl trisulphide (7.05-14.95%), methyl propyl disulphide (9.5%), propyl *trans*-propenyl disulphide (7.87%), allyl propyl disulphide (3.56%), dipropyl tetrasulphide (3.04%), dimethyl trisulphide (1.14-16.64%), propyl *cis*-propenyl disulphide (4.67-9.72%), dimethyl disulphide (1.31%), dimethyl tetrasulphide (0.46-7.24%), and isopropyl disulphide (0.31%)). The water-insoluble extractive obtained from onion EO consists of a complex mixture of volatile sulfur compounds, mostly mono-, di-, tri-, and tetrasulphides with different alkyl groups [15].

Although EOs are available as natural flavoring, they have some limitations that must be overcome before applying them to food systems. The main properties that make EOs difficult to apply in the food system are low water solubility, high volatility [16, 17], and strong odor [16]. The hydrophobicity properties of EOs cause nonuniform distribution in food matrices and reduce their antimicrobial effectiveness when directly incorporated into foods due to their hydrophobic binding with food components [18]. Also, a high concentration of EOs affects the organoleptic properties because the concentration of essential oils required to cause a bacterial inhibitory effect in vitro is significantly higher than the concentrations required to cause similar effects in real foods [19]. Hence, the emulsification process can help to solve this problem [20].

Emulsions or dispersions are produced by homogenizing two immiscible phases together in the presence of stabilizer molecules [21, 22]. Emulsification of EOs not only able to overcome the limitations of EOs but also able to increase their antibacterial activity. The emulsion-based systems are the most desirable delivery systems for encapsulating, protecting, delivering, and releasing poorly oil- or water-soluble drugs and food-active ingredients [23]. Through the emulsification process, the dispersed phase is broken up into small droplets [24]. It might facilitate diffusion of the encapsulated antibacterial to reach the right site (i.e., membrane of bacteria). Additionally, the emulsions might cause the permeabilization of the cells and disrupt the bacteria's cell membrane integrity [25]. According to Topuz et al. [26], the emulsified EO of anise showed better and longer-term physicochemical stability and antimicrobial activity compared to bulk anise oil. Moreover, emulsified EOs also showed higher antimicrobial activity, even at far lower concentrations [27]. Furthermore, food products during storage and processing undergo various environmental stresses such as sunlight, heat, and freezing. Therefore, there is an increasing emphasis on developing a more fundamental understanding of the influence of conditions and environmental stresses on the functionality of the stabilizer system [22] and, as a result, on the stability of the emulsion system. To the best of our knowledge, the antibacterial activity and physical properties of onion oil in water emulsion as a food flavoring agent and the effect of environmental stress on the physical properties and antibacterial activity of onion oil in water emulsion have not been studied. For the stability of emulsions, sodium caseinate was selected as an emulsifier agent since not only it is frequently used as a natural emulsifier [28] but also, in the food industry, it is one of the proteins that is largely used as an ingredient [29]. Sodium caseinate is flexible, moderately highly soluble, and quickly adsorbed at the oil-water interface [28]. Thus, this study is aimed at determining the antibacterial activity against food-associated bacteria and physical properties of the onion essential oil in water emulsion and at evaluating the effect of environmental stress, namely, thermal, UV, and freeze-thaw treatment, on the physical properties and antibacterial activity of the best-produced onion oil emulsion.

## 2. Materials and Methods

### 2.1. Materials

The pure essential oil of onion (*Allium cepa* L.) was provided by BF1 Soul Brand, Malaysia. Sodium caseinate was obtained from R&M chemicals (Tamil Nadu, India). Acetone was purchased from ACME Chemicals (Malaysia, Selangor). Mueller Hinton Broth (MHB) and Mueller Hinton Agar (MHA) were supplied by Merck (Berlin, Germany). The blank paper disk and sterile swab used for antimicrobial properties were purchased from Bioeconomy Co. (Kuala Lumpur, Malaysia). *Listeria monocytogenes* ATCC 19114, *Staphylococcus aureus* ATCC 25923, *Salmonella* Typhimurium ATCC 19585, and *Escherichia coli* ATCC 25922 were the target bacteria.

### 2.2. Fabrication of Onion Essential Oil Emulsion

The onion EO emulsions were prepared according to the solvent displacement technique reported by Ribeiro et al. [30]. The organic phase was prepared by dissolving 5% (*w*/*w*) onion essential oil in 5% (*w*/*w*) acetone. The aqueous phase was prepared by dissolving sodium caseinate (3, 5, and 7% *w*/*w*) into distilled water (87, 85, and 83% *w*/*w*). The selected concentrations of sodium caseinate were based on our preliminary study. Sodium caseinate at concentrations of 1 and 2% (*w*/*w*) did not form stable emulsions (i.e., it showed creaming after a few hours). The organic phase was added to the aqueous phase that had been hydrated for 24 hours under moderate magnetic stirring (1500 rpm) and continuous magnetic stirring. Finally, the resulting emulsion was subjected to rotary evaporation (Eyela NE-1101, Tokyo Rikakikai Co., Ltd., Tokyo, Japan) at a temperature of 40°C under reduced pressure (0.25 bar) to remove the organic solvent (acetone), which was added at an earlier stage. 5% (*w*/*w*) of distilled water was added to each fabricated formula. All the procedures had been done aseptically. The formulation of onion EO emulsions with different concentrations of sodium caseinate is shown in Table 1.

### 2.3. Physical Properties

#### 2.3.1. Average Droplet Size Measurement

An optical microscope with a 40x objective lens and a 10x eyepiece was used to capture the images of the emulsions produced. The measurement of the droplet size was taken by the software LOGO (size 240 × 60) installed on an Android tablet attached to the optical microscope. Hence, the measurement of the droplet size was recorded as an image with three replications. The droplet size measurement was performed in triplicate for each sample.

#### 2.3.2. Stability (Ke) Measurement

The centrifugal acceleration method was used for the evaluation of the emulsion according to He et al. [31] with slight modification. Briefly, 5 g of the emulsion was transferred into a 15 ml centrifuge tube and underwent centrifugation (Hettich, Universal 32 centrifuge, Germany) for 10 minutes at 4000 rpm (~2365 g). After centrifuging, the emulsion layer was transferred into a 20 ml universal bottle with a slow and steady motion. Then, the emulsion was diluted with distilled water in a ratio of 1 : 5. The absorbance value of the diluted samples was determined by a spectrophotometer (Thermo Fisher Scientific, GENESYS 20, CA, USA) at a wavelength of 500 nm. The constant of centrifugal stability (Ke) was calculated according to the following formula:

(1) $$\begin{array}{c} \text{Ke}=\frac{\left|{\text{A}}_{0}-\text{A}\right|}{{\text{A}}_{0}}\times 100\%, \end{array}$$

where *A*_0_ is the absorbance of the emulsion before it is diluted and *A* is the absorbance of the emulsion after centrifugation. The stability assay was performed in triplicate for each sample.

#### 2.3.3. Turbidity Measurement

The turbidity of emulsions was measured according to Komaiko [32] and Taghavi et al. [33] with little modification. In this regard, a UV-visible spectrophotometer (Thermo Fisher Scientific, GENESYS 20, CA, USA) at 600 nm was used. The emulsion of onion was served with distilled deionized water (sample: distilled water, 1 : 5). The distilled deionized water was considered as a blank. Turbidity measurements were performed in triplicate.

#### 2.3.4. Color (Lightness and Chroma) Measurement

The color of the emulsions was measured instrumentally using a Hunter Lab colorimeter (Konica Minolta Sensing, Inc., Osaka, Japan), and the results were expressed in terms of *L*∗ (lightness), *a*∗ (redness), and *b*∗ (yellowness) (CIELAB system). The instrument is calibrated by using black and white tiles. Color parameters were expressed in Hunter Lab units. The *L*∗ represents lightness, where low *L*∗(0) is dark (black) and high *L*∗(100) is light (white). Color intensity was characterized by chroma by using the following formula [34]:

(2) $$\begin{array}{c} C={\left(a{*}^{2}+b{*}^{2}\right)}^{1/2} \end{array}$$

The color measurement was carried out in triplicate.

### 2.4. Antibacterial Activity

#### 2.4.1. Preparation of Bacterial Cultures

For the preparation of bacterial culture, one to five colonies were transferred into a sterile universal bottle containing MHB and incubated overnight at 37°C for 24 hours. Then, the overnight culture was diluted with MHB to 0.5 McFarland standard and checked with a visible spectrophotometer (Thermo Fisher Scientific, GENESYS 20, CA, USA). The turbidity of the 0.5 McFarland standard solution was 0.132 OD at 600 nm [33, 35]. The viability of 0.5 McFarland standard solution for each strain was approximately equal to 10^8^ cells per millilitre.

#### 2.4.2. Agar Disk Diffusion Assay

The agar disk diffusion (ADD) test/assay was performed for the measurement of clear zone inhibition according to the methods reported by Choo et al. [36], with minor modifications. Briefly, the 0.5 McFarland culture was diluted at 1 : 100. Then, the 100 *μ*l inoculum was spread evenly on MHA in a Petri dish using a sterile cotton swab. Paper disks containing positive control, negative control, and EO emulsion were placed on the surface of the agar. 40 *μ*l of each formulation of emulsion, control negative, and bulk EO pipetted on the paper disks. The distance between disks is no closer than 24 mm from center to center. The plates were incubated at 37°C for 24 hours. The zone of inhibition was measured by using a sliding calliper after 24 hours of incubation and recorded as millimeter (mm). The results represented the net zone of inhibition, including the diameter (6 mm) of the paper disk. The positive and negative controls were streptomycin and 10% *w*/*w* dimethyl sulfoxide (DMSO), respectively. Also, bulk EO was dissolved in 10% (*w*/*w*) DMSO. 10% (*w*/*w*) DMSO was sterilized by filtration through a 0.2 *μ*m membrane filter. The experiment was done in three replicates.

### 2.5. Environmental Stress

The best sample among the three formulations in terms of physical properties and antibacterial activity undergoes environmental stress. Thermal processing (pasteurization time and temperature), UV radiation, and freeze-thaw cycle were selected as the environmental stresses.

#### 2.5.1. Thermal Processing Treatment

Water bath heating stability was evaluated according to Galvão et al. [37]. The universal bottles containing the emulsions were incubated in a water bath at 63°C for 30 min. The samples were subsequently cooled at room temperature for 30 min. The physical and antibacterial properties of the emulsion were evaluated after thermal processing treatment.

#### 2.5.2. UV Radiation Treatment

The UV radiation treatment was done according to Sheng et al. [38] with modification. EO emulsion was transferred into a small beaker and placed under the UV light of a laminar flow hood (CFM series, ERLA, ERLA Technologies (M) Sdn. Bhd. Malaysia) for 2 hours at room temperature. The physical and antibacterial properties of the emulsion were evaluated after UV radiation treatment.

#### 2.5.3. Freeze-Thaw Treatment

The environmental study of thermal processing was conducted according to Aoki et al. [39] with modification. The EO emulsion was transferred into the universal bottle and incubated in a -18°C freezer for 24 hours. Then, thaw by incubating in a water bath at 30°C for 2 hours. The physical and antibacterial properties of the emulsion were evaluated after freeze-thaw treatment.

### 2.6. Statistical Analysis

The data were analyzed by one-way analysis of variance (ANOVA). Fisher's multiple comparison test at a confidence level of *p* ≤ 0.05 was applied to find any significant difference among the samples. Statistical analyses were performed with the Minitab version 16 package (Minitab 16, Minitab Inc., State College, PA).

## 3. Results and Discussion

### 3.1. Physical Properties of Onion EO Emulsion

**Figure 1:**
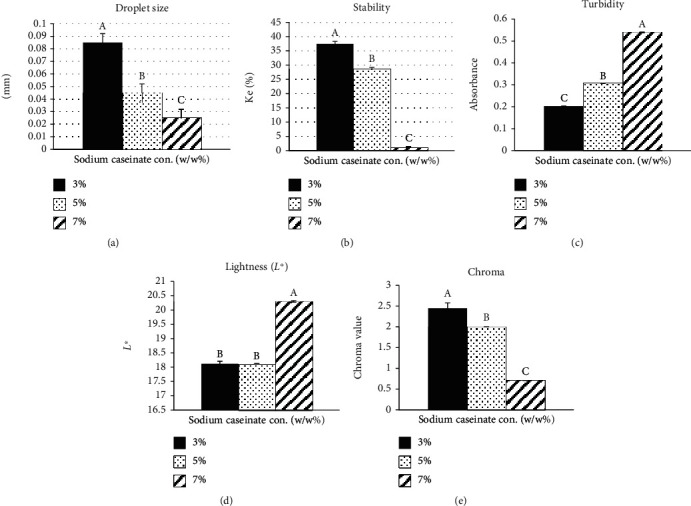
The effect of various concentrations of sodium caseinate as an emulsifier on droplet size (a), emulsion stability (b), turbidity (c), lightness (d), and chroma (e). Values are means ± standard deviations (*n* = 3). Different letters show statistically significant differences between values (*p* < 0.05). con.: concentration.

Figure 1(a) presents the average droplet size of onion emulsion (OE) fabricated with various concentrations of sodium caseinate. Data analysis showed that the concentration of sodium caseinate had a significant (*p* < 0.05) effect on droplet size. The droplet size of the OE ranged from 0.085 to 0.025 mm. The largest droplet size was obtained with 3% (*w*/*w*) sodium caseinate. The 7% (*w*/*w*) concentration of sodium caseinate showed the lowest droplet size compared to the 3 and 5% (*w*/*w*) concentrations of sodium caseinate. Overall, the chart (Figure 1(a)) shows that there has been a decrease in the droplet size by increasing the concentration of sodium caseinate. These results probably occurred due to sufficient concentrations of the surfactant, which were enough to fully cover the newly formed droplets of oil and rapidly adsorb at the interface [40]. Previous studies by Srinivasan et al. [41] have shown that the increase in sodium caseinate decreases the size of droplets. Higher protein concentration in the aqueous phase improves the availability of emulsifier to encapsulate oil droplets; eventually, smaller droplet size was produced [42].

The stability of OEs was tested by using the centrifugal acceleration method (Figure 1(b)). The lowest stability value recorded as the most stable emulsion. High stability emulsion can resist coalescence [43]. The stability study showed that concentration had a significant (*p* < 0.05) effect on the stability of the emulsion. OEs containing 7 (1.21%) and 3% (*w*/*w*) (37.47%) sodium caseinate were the most and least stable emulsions, respectively. In Figure 1(b), there is a clear trend of increasing stability by increasing emulsifier concentration. Thus, emulsions prepared by emulsifiers with higher concentrations are more resistant to coalescence. Coalescence of emulsions is an irreversible process by which two or more droplets merge during contact to form a single droplet. This reduction of the average droplet size causes an increment in the stability of the emulsion [43]. This finding is in agreement with Xue [43], who has proven that a higher concentration of emulsifiers is more stable than a lower concentration. Another possible explanation is that the larger droplets appeared to be surrounded by many smaller droplets and other small structures. This can be attributed to the fact that large droplets tend to cream faster and that droplets in the cream layer tend to pack closely together, which may facilitate coalescence that can cause the emulsion to reduce its stability [32, 44]. Thus, increasing the concentration of the emulsifier can reduce the droplet size of the emulsion stability.

The optical properties of the emulsion-based delivery systems are particularly important in terms of their practical application. In some applications, it is important to have an optically transparent delivery system (e.g., clear beverages), whereas it may not be important in some other cases (e.g., opaque food). It may even be desirable to have a turbid delivery system (e.g., cloudiness in soft drinks). In most cases, the emulsion is prepared by aiming to have a much smaller droplet and a clear appearance. However, it may not be possible to make a highly clear nanoemulsion in some cases. This issue is more obvious if the emulsion contains a high concentration of oil compound and a large droplet size. Optical properties including turbidity (Figure 1(c)), lightness (Figure 1(d)), and chroma (Figure 1(e)) of the OE in our study were determined. The results indicated that the concentration of sodium caseinate had a significant (*p* < 0.05) effect on turbidity, lightness, and chroma. The OE containing 7% (*w*/*w*) emulsifier had the highest turbidity (0.54) and lightness (20.3) but the lowest chroma (0.71) among the other fabricated samples. Also, the OE containing 3% (*w*/*w*) emulsifier had the lowest turbidity (0.203) and lightness (18.11), but the highest value of chroma (2.45) among the other fabricated samples.

As aforementioned earlier, sodium caseinate concentration had a significant (*p* < 0.05) effect on turbidity. Increasing the concentration of sodium caseinate as an emulsifier causes an increase in turbidity. This observation could be due to the fact that the turbidity of an emulsion system is mainly governed by the concentration level of the dispersed oil phase and emulsifier content [45]. This finding is in agreement with the previous researcher. They found that the concentration of gum Arabic influenced the turbidity of oil-in-water (o/w) emulsions stabilized with gum Arabic [46].

As shown in Figures 1(d) and 1(e), lightness and chroma had opposite trends with each other. It means that by increasing the concentration of sodium caseinate, lightness and chroma are increased and decreased, respectively. Also, Chung and McClements [34] stated that color intensity is usually inversely related to the lightness. This might be due to the fact that the chromaticness of the emulsions decreased with increasing droplet concentration (i.e., *a*∗ and *b*∗ tended toward zero), which is to be expected because the magnitudes of *a*∗ and *b*∗ are approximately proportional to the reciprocal of the lightness. So that, an increase in lightness tends to cause a decrease in chromaticity [47].

### 3.2. Antibacterial Properties of Onion EO Emulsion

The antibacterial activity of bulk onion EO as a target against four different bacteria, namely, *Escherichia coli*, *Salmonella* Typhimurium, *Staphylococcus aureus*, and *Listeria monocytogenes*, was assessed with the disk diffusion method. As results are presented in Table 2, the inhibition zone of the target bulk EO was 6.17 to 7.25 mm against *S.* Typhimurium and *L. monocytogenes*, respectively. In addition, no inhibition zones were produced by a control negative (10% DMSO), and there was no antibacterial activity for *E. coli* and *S. aureus*. Therefore, *S.* Typhimurium and *L. monocytogenes* were selected for the next step of our evaluation of the antibacterial activity.

**Figure 2:**
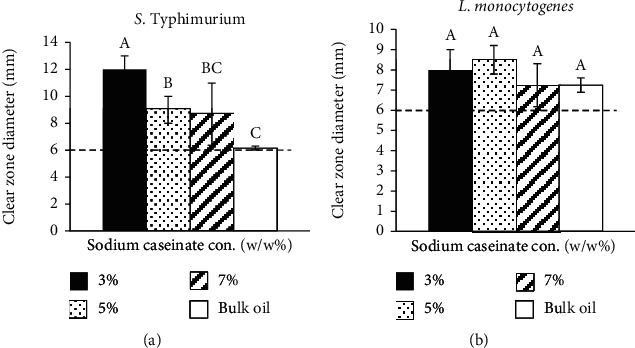
The effect of various concentrations of sodium caseinate as an emulsifier on clear zone diameter (mm) of S. Typhimurium (a) and L. monocytogenes (b). Values are means ± standard deviations (*n* = 3). Inhibition zone includes diameter of disc (6 mm). Different letters show statistically significant differences between values (*p* < 0.05).

The antibacterial activity of fabricated emulsions containing various concentrations of sodium caseinate as an emulsifier against *S.* Typhimurium and *L. monocytogenes* as target bacteria was tested via the disk diffusion method. The results are presented in clear zone diameters in Figure 2. The inhibition zone of the emulsified onion EO ranged from 12.00 to 7.25 mm. No inhibition zones were produced by a control negative (10% DMSO; emulsions contain no EO). As aforementioned earlier, 5% (*w*/*w*) onion essential oil in free form and streptomycin considered control positive. The lowest zone inhibition diameters for *S.* Typhimurium and *L. monocytogenes* belonged to OE, which contained 7% (*w*/*w*) sodium caseinate at 8.67 and 7.25 mm. The highest zone inhibition diameters for *S.* Typhimurium and *L. monocytogenes* belonged to OE containing 3% (*w*/*w*) (12.00 mm) and 5% (*w*/*w*) (7.25 mm) sodium caseinate, respectively.

Generally, the antibacterial activity of onion EO in emulsified forms is increased, except OE in emulsion contains 7% (*w*/*w*) sodium caseinate against *L. monocytogenes* that is maintained constant compared to onion bulk oil. In other words, there is an improvement in antibacterial activity after the emulsification process. The increase in antibacterial activity might be due to the increase of EO dispersibility, which enhances the antibacterial activity [48]. Overall, the OE containing 7% (*w*/*w*) sodium caseinate showed the lowest antibacterial activity among all OEs. It might be due to the fact that the emulsion system may limit the contact of the antibacterial compound with the membrane of bacteria [49] since oil is covered by a higher concentration of sodium caseinate; however, this increment was not significant (*p* > 0.05) against *L. monocytogenes* and 5% (*w*/*w*) sodium caseinate against *S.* Typhimurium. Hence, by suitable partitioning between the aqueous and a lipid phase as well as proper concentration of emulsifier, the solubility of hydrophobic antibacterial might be improved, and consequently, the inhibitory activity and bactericidal activity might be enhanced.

Among all fabricated emulsions with different concentrations of emulsifier, OE containing 7% (*w*/*w*) sodium caseinate showed lower droplet size and chroma with higher stability, turbidity, and lightness. It also has good antibacterial activity against all selected target bacteria. Therefore, it was selected as the best formulation in terms of physical properties and antibacterial activity. Hence, the further step of this study (i.e., environmental stress section) was continued with OE containing 7% (*w*/*w*) sodium caseinate.  Table 3 shows the logic of OE contained 7% (*w*/*w*) selection as the best sample.

### 3.3. Effect of Environmental Stress on Physical Properties of Onion EO Emulsion

**Figure 3:**
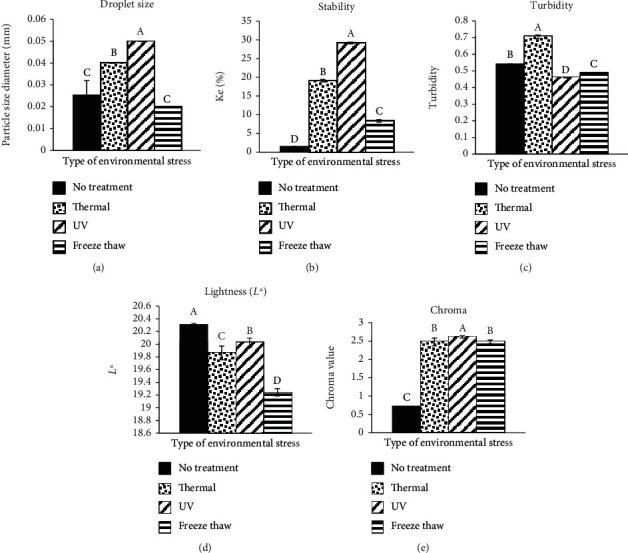
The effect of environmental stress (thermal processing, UV radiation, and freeze-thaw) on droplet size (a), emulsion stability (b), turbidity (c), lightness (d), and chroma (e) on the best selected emulsified sample (emulsion contained 7% (*w*/*w*) sodium caseinate). Values are means ± standard deviations (*n* = 3). Different letters show statistically significant differences between values (*p* < 0.05).

The best OE, which contained 7% (*w*/*w*) sodium caseinate, underwent the environmental stress treatment to characterize its physical properties (Figure 3). The best OE (BOE), which contained 7% (*w*/*w*) sodium caseinate, was considered the control in this section of our study. As shown in Figure 3(a), the droplet size of the BOE after undergoing thermal processing and UV radiation increased significantly (*p* < 0.05) whereas there were no significant (*p* > 0.05) changes for the sample under freeze-thaw treatment. The UV and freeze-thaw treatments had the largest and smallest droplet sizes, respectively. In other words, among the environmental stress treatments, UV radiation treatment shows the highest droplet size (0.05 mm) compared to thermal and freeze-thaw treatment (0.04 and 0.02 mm, respectively). Also, the stability of the BOE after undergoing all types of environmental stress decreased significantly (*p* < 0.05) as shown in Figure 3(b). The highest emulsion stability was due to freeze-thaw (8.347%), and the lowest emulsion stability was due to UV radiation (29.173%).

These obtained trends from droplet size and stability of BOE illustrated that it becomes less stable with larger droplet size (except in freeze-thaw treatment) after environmental stress. It might be due to the emulsion being less stable when the temperature of the emulsion was about 30°C [50]. Furthermore, in the study that had been done by Euston and Mayhill [51], sodium caseinate decreased in casein surface coverage on the emulsion above 15°C. Lower emulsifier surface coverage can result in lower emulsion stability.

On the other hand, freezing also promotes the destabilization of emulsions. Freezing causes ice crystal formation which forced oil droplet closer together and insufficient free water to maintain the emulsion molecule and increases the concentration in the nonfrozen aqueous phase surrounding the oil droplet; ice crystal penetrates and disrupts the interfacial membrane surrounding the oil droplets leading to the coalescence of the droplets during thawing; fat phase within droplet solidifies, and protruding fat crystals may penetrate and disrupt the interfacial membrane surrounding the oil droplets [52].

Furthermore, UV is able to cause the peptide in the emulsifier to change. A molecule containing *α*-helices has a high hydrophobic moment, which contributes to good emulsifying properties [53]. Genuino et al. [54] showed a similar result in their research. They found that UV irradiation reduced the stability of emulsions. In addition, thermal treatment (heat energy) caused the movement of droplets in the emulsion and, subsequently, increased the possibility of collusion of droplets in the emulsion system. It might be affected by both increasing the droplet size and instability in the thermally treated emulsion due to instability mechanisms.

The optical properties of an emulsion-based delivery system are essential in order to predict the visual properties of its application, especially during storage. Optical properties (i.e., turbidity, lightness, and chroma) of the BOE were measured (Figures 3(c)–3(e)). The environmental stresses significantly (*p* < 0.05) affect turbidity. Emulsion with thermal treatment had the highest turbidity (0.706), while UV treatment (0.459) and freeze-thaw (0.487) had less turbidity after the treatment. The increment of turbidity in the case of thermal processing stress might be due to the effect of the solubility of the dispersed phase in the continuous phase causing an increase in the absorbance, whereas increasing temperature was not applicable for UV radiation and freeze-thaw cases.

Moreover, as shown in Figures 3(d) and 3(e), the environmental stresses significantly (*p* < 0.05) affect lightness (*L*∗) and chroma. The highest and lowest lightness among all environmental stress treatments belongs to UV (20.027) and freeze-thaw (19.243) treatments, respectively, while the highest and lowest chroma values belong to UV (2.617) and thermal (2.473) treatments, correspondingly, although statistical analysis did not show a significant difference among thermal and freeze-thaw treatments. From the trend of lightness and chroma, it can be clearly found that their trends are opposite of each other. It means that environmental stress decreases lightness while the chroma for a similar situation is increased. Environmental stress might decrease the droplet concentration, homogeneity, and dispersibility. Therefore, a decrease in lightness tends to cause an increase in chromaticity (as discussed earlier in Section 3.1).

### 3.4. Effect of Environmental Stress on Antibacterial Properties of Onion EO Emulsion

**Figure 4:**
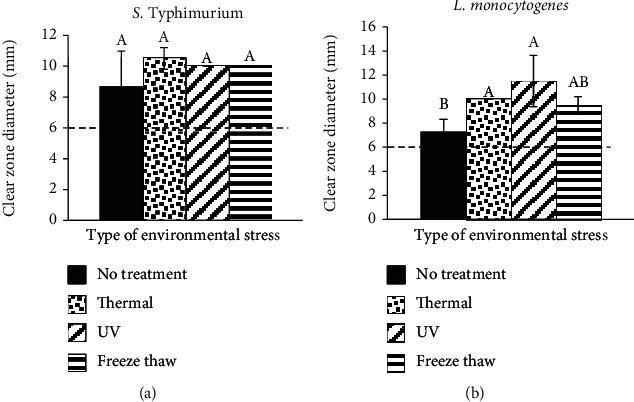
The effect of various environmental stress on clear zone diameter (mm) of S. Typhimurium (a) and L. monocytogenes (b). Values are means ± standard deviations (*n* = 3). Inhibition zone includes diameter of disc (6 mm). Different letters show statistically significant differences between values (*p* < 0.05).

The antibacterial activity of BOE after undergoing the environmental stress treatments against two selected bacteria was investigated through the disk diffusion method (Figures 4(a) and 4(b)). The inhibition zone of the BOE after environmental stress treatments changed from 9.50 to 11.50 mm. Thermal and UV radiation treatments caused the creation of the highest inhibition zone diameter for *S.* Typhimurium and *L. monocytogenes*, respectively. Overall, BOE after environmental stress treatments showed better antibacterial activity against both bacteria, although these increments were not significant (*p* > 0.05). A possible explanation for this might be that the antibacterial EO was released from the encapsulated antibacterial emulsion, which happened due to exposure to environmental stress treatments. Hence, the free form of the EO in the stressed system, while spread homogeneously compared to bulk EO, might be the reason for the increment in the antibacterial activity compared to control (BOE).

## 4. Conclusions

This study was conducted with the purpose of determining the physical and antibacterial properties of onion oil as a food flavoring agent under normal and environmental stress conditions loaded in emulsion with different concentrations of sodium caseinate as an emulsifier. The emulsification of onion EO revealed that by increasing the concentration of emulsifier, the droplet size and stability of emulsions decrease and increase, respectively. Also, emulsification of onion oil improved the antibacterial activity of onion oil against target bacteria. Emulsion with 7% (*w*/*w*) sodium caseinate showed the best physical properties and antibacterial activity. Therefore, an emulsion with 7% (*w*/*w*) sodium caseinate was subjected to studies to determine the effect of environmental stress on it. Under thermal, UV radiation, and freeze-thaw stress treatments, the emulsion system showed an increase in the droplet size and decreased stability. Moreover, stress treatments of BOE presented better antibacterial activity compared to BOE with no treatment and bulk oils as controls. Further investigation to understand the antibacterial mechanisms of nanodelivery using nanoemulsion as a carrier should be explored. In this regard, optimization of process parameters should be considered for better efficiency. Moreover, further research is recommended to study the antifungal inhibitory activity of the emulsified and free forms of onion oil while it can be examined in a real food system in both fresh and stored food under environmental stress and to investigate the sensory effect via a panel of sensory evaluation.

## Figures and Tables

**Table 1:** The formulation of onion EO emulsions with different concentrations of sodium caseinate.

Formulation	Onion oil (*w*/*w* %)	Emulsifier (sodium caseinate, *w*/*w* %)	Acetone (*w*/*w* %)	Distilled water (*w*/*w* %)
Formulation 1	5	3	5	87
Formulation 2	5	5	5	85
Formulation 3	5	7	5	83

**Table 2:** The clear zone inhibition diameter (mm) of onion essential oil against selected bacteria.

Bacteria	Onion oil (5% of bulk oil)	Streptomycin
*E. coli*	6.00 ± 0.000^b^	10.90 ± 0.81^b^
*S.* Typhimurium	6.17 ± 0.144^b^	11.00 ± 0.22^b^
*S. aureus*	6.00 ± 0.000^b^	12.29 ± 0.61^a^
*L. monocytogenes*	7.25 ± 0.354^a^	12.2 ± 0.327^a^

Note: values are given as mean ± standard deviation (*n* = 3). Inhibition zone includes diameter of disc (6 mm). Different letters indicate significant differences within columns at *p* < 0.05.

**Table 3:** The sample order based on the top to poor desirability.

Responses	The most desirable sample order
Droplet size (mm)	7% *w*/*w* SC OE > 5%*w*/*w* SC OE > 3%*w*/*w* SC OE
Physical stability (%)	7% *w*/*w* SC OE > 5%*w*/*w* SC OE > 3%*w*/*w* SC OE
Turbidity	3% *w*/*w* SC OE > 5%*w*/*w* SC OE > 7%*w*/*w* SC OE
Color (*L*^∗^)	7% *w*/*w* SC OE > 5%*w*/*w* SC OE = 3%*w*/*w* SC OE
Chroma	7% *w*/*w* SC OE > 5%*w*/*w* SC OE > 3%*w*/*w* SC OE
Antibacterial activity against *S.* T	3% *w*/*w* SC OE > 7%*w*/*w* SC OE = 5%*w*/*w* SC OE
Antibacterial activity against *L. m*	7% *w*/*w* SC OE = 5%*w*/*w* SC OE = 3%*w*/*w* SC OE

Note: SC: sodium caseinate; OE: onion emulsion; *S.* T: *S.* Typhimurium; *L. m*: *L. monocytogenes*.

## Data Availability

Data will be available upon request.
